# Microbiological, Physicochemical, and Immunological Analysis of a Commercial Cashew Nut-Based Yogurt

**DOI:** 10.3390/ijms21218267

**Published:** 2020-11-04

**Authors:** Christopher P. Mattison, Kayanush J. Aryana, Kristen Clermont, Eric Prestenburg, Steven W. Lloyd, Casey C. Grimm, Richard L. Wasserman

**Affiliations:** 1 Southern Regional Research Center, United States Department of Agriculture, Agricultural Research Service, New Orleans, LA 70124, USA; kristen.clermont@usda.gov (K.C.); eric.prestenburg@usda.gov (E.P.); steven.lloyd@usda.gov (S.W.L.); casey.grimm@usda.gov (C.C.G.); 2 School of Nutrition and Food Science, Louisiana State University Agricultural Center, 115 Dairy Science Building, Baton Rouge, LA 70803, USA; karyana@agcenter.lsu.edu; 3 Oak Ridge Institute for Science and Education, U.S. Department of Energy, Oak Ridge, TN 37831-0117, USA; 4 Allergy Partners of North Texas Research, Dallas, TX 75230, USA; drrichwasserman@gmail.com

**Keywords:** cashew, allergen, yogurt, lactic acid bacteria, fermentation, food allergy

## Abstract

Nut-based milks and yogurts are gaining popularity, but may not offer the same benefits as dairy yogurts to consumers**.** Cashew nuts often cause severe allergic reactions, and cashew nut allergens are stable to several types of processing. To compare its characteristics to dairy yogurt and characterize the effects of fermentation on the Ana o 1–3 cashew nut allergens, a commercial yogurt made from cashew nuts (Cashewgurt) was evaluated for microbiological, physiochemical, and immunological properties. Average counts for lactobacilli and *Streptococcus thermophilus* were greater than 10 million colony forming units per milliliter, indicating the capacity to provide a health benefit. Cashewgurt pH and viscosity values were comparable to cow milk yogurts, and it was off white in color. SDS-PAGE analysis indicated a clear reduction in Ana o 1 and 2, and immuno-assay with polyclonal anti-cashew IgG antibody and cashew-allergic IgE indicated an overall reduction in allergen content. In contrast, SDS-PAGE, mass spectrometry, immunoblot, and ELISA all revealed that Ana o 3 was relatively unaffected by the fermentation process. In conclusion, Ana o 1 and Ana o 2 are sensitive to degradation, while Ana o 3 survives lactic acid bacterial fermentation during yogurt production. The analysis presented here indicates that cashew nut yogurt is not suitable for those with cashew nut allergy.

## 1. Introduction

Food processing methods including physical, mechanical, and enzymatic manipulations can enhance the nutritional, sensory, storage, or safety qualities of foods. Microbial fermentation has been used for centuries to produce a wide variety of nutritious and delicious foods including cheeses, sauerkraut, kefir, kimchi, yogurt, wine, and beer. Fermented foods offer numerous health benefits associated with the bioactive metabolites and peptides produced by microbial metabolism during food production or in the human gut during digestion [1].

Lactic acid bacteria (LAB) are Gram-positive, non-spore-forming rods normally found in the human mouth and intestinal tract. They are commonly used as probiotics. Food fermentation by LAB has long been a popular method to produce foods with high nutritional value and sought-after sensory qualities. LAB have evolved proteolytic systems to break down proteins in their environment to obtain essential amino acids [2]. Although the nutritional demands of *Lactobacillus plantarum* (*L. plantarum*) are greater than some other species, it is by far the predominant species used for plant-based fermentations and has recently been used to ferment substrates such as quinoa and wheat slurry [3]. Comparative genomic studies of LAB such as *L. plantarum* and *Streptococcus thermophilus* have provided clues to understanding the proteolytic systems these bacteria harbor and the differences in peptidase families, peptide transport systems, and cell wall-associated proteinases [2], leading to a diversity of fermentation products.

Fermentation can also result in the production of bioactive bacterial metabolites. Short-chain fatty acids (SCFAs) such as acetate and butyrate are produced by fermentation of dietary fiber and have been associated with regulation of the immune system [4]. For example, altered gut microbiota and increased acetate production have been linked to the promotion of improved glucose-stimulated insulin secretion and a reduction in metabolic syndrome [5]. The influence of gut microbes and their metabolites on the regulation of the intestinal environment, immune system, and other human health factors is a burgeoning area of investigation.

Food allergy results in a reduced quality of life for food-allergic patients and their families. Several factors are thought to contribute to the increase in reported food allergy in recent years. More recently, our understanding and appreciation of the important role gut bacteria play in mediating tolerance to foods has increased [6]. The presence of certain bacteria, as well as the bioactive metabolites they generate, are thought to modulate intestinal and immunological responses to the foods we eat. For example, fermentation of tree nuts including almonds, hazelnuts, pistachios, and walnuts in vitro using human fecal bacteria produces beneficial SCFAs [7]. Further, fermentation of milk and soy can reduce IgE antibody binding to allergens [8].

Cashew nuts are produced in several regions around the world, and the FDA considers foods such as cashew nuts to be part of a “heart-healthy” diet. Cashew nuts are rich in beneficial lipids, protein, micronutrients, and bioactive compounds. However, cashew nuts contain three conserved seed storage proteins (Ana o 1, Ana o 2, and Ana o 3) that have defined IgE-reactive epitopes [9,10,11] that can cause severe allergic reactions. Cashew nuts undergo several heating and drying steps during industrial processing [12]. Cashew nut allergen solubility can be modified by heating and a heating-induced modification of Ana o 3 has been observed [13,14]. Overall, cashew nut allergens have proven to be relatively resistant to many types of physical, chemical, and enzymatic processing methods [8,15,16]. Proteolytically processed cashew nut extracts, however, have been demonstrated to be hypoallergenic in a mouse model [17]. The objective of this study is to characterize the microbiological and physiochemical properties as well as the allergen content of a commercial yogurt product made from cashew nuts.

## 2. Results

### 2.1. Microbiological and Physicochemical Properties of Cashewgurt

The average counts for lactobacilli, bifidobacteria and *S. thermophilus* in Cashewgurt are shown in Table 1. Both lactobacilli and *S. thermophilus* had more than 10^7^ CFU/mL. The coliform counts, yeast and mold counts were <10, implying that the Cashewgurt had no post-pasteurization contamination.

The average pH (Table 1) for the Cashewgurt is comparable to that measured for cow milk yogurt (4.40) [18]. The average TA expressed as percent lactic acid of Cashewgurt (Table 1) was low in comparison to cow milk yogurt (1.24 %) [19]. The average viscosity (Table 1) of the Cashewgurt was comparable to cow milk-based yogurt (13098 cP) [18].

The average L* (light/dark) value obtained for the Cashewgurt was 84.67 ± 0.47, indicating that the Cashewgurt was off white in color. Reported L* values for cow milk yogurt are higher (94.47 ± 0.10) [20]. The average a* (red / green) value obtained was 0.73 ± 0.15, indicating a very slight red. In comparison, cow milk yogurts had a negative a* value (−2.03 ± 0.08) [20]. The average b* (yellow / blue) value obtained was 8.89 ± 0.53, indicating a slight yellowness. Cow milk yogurts were reported to have a higher b* value (12.15 ± 0.13) [20]. The average C* (color intensity) value obtained for the Cashewgurt was 8.92 ± 0.52, indicating that the color intensity is very slight. The average h* (hue angle) value was 85.23 ± 1.18, indicating that the Cashewgurt color falls into a space blending a little red and relatively more yellow.

### 2.2. Cashewgurt Soluble Protein

**Figure 1:**
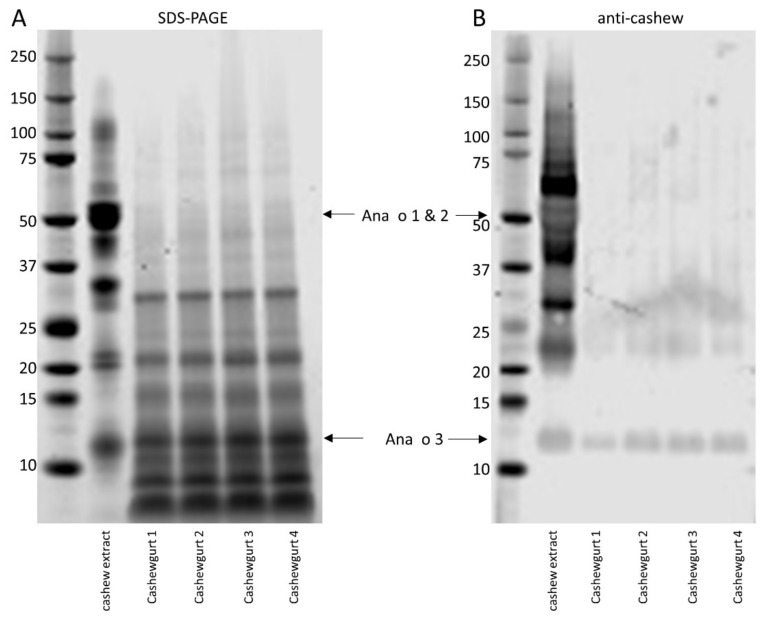
Comparison of soluble cashew nut extract and Cashewgurt protein. Non-reducing SDS-PAGE analysis of soluble cashew nut and Cashewgurt protein extracts (**A**) and immunoblot of identical material with a rabbit polyclonal antibody (**B**). Ana o 1, 2, and 3 are indicated with arrows and molecular weight standards are shown to the left of each panel.

Phosphate-buffered saline was mixed 1:1 with Cashewgurt to isolate soluble protein from four independent samples. Non-reducing SDS-PAGE indicated that the soluble protein in Cashewgurt samples had a distinctly different migration pattern compared to unfermented cashew nut extract (Figure 1). Many of the higher-molecular-weight proteins, including Ana o 1 and Ana o 2, were absent or only faintly observed in the yogurt samples, suggesting that they were at least partially digested. However, the Cashewgurt sample contained four distinct bands migrating just above and just below the 10 kDa marker. These bands were excised and subjected to trypsin digestion, peptide extraction, and LC–MS/MS analysis for protein identification. The top band in this group migrated at a rate similar to Ana o 3 from the cashew nut extract, and immunoblot with rabbit polyclonal anti-cashew antibody also recognized a band migrating at an identical position with Ana o 3 in cashew nut extract (Figure 1). Further, three peptides matching Ana o 3 sequence were observed via LC–MS/MS analysis of this band excised from gels, confirming the presence of Ana o 3. Ana o 3-specific peptides were also observed in two of the faster migrating bands near the 10 kDa marker, indicative of some level of Ana o 3 degradation or conformational instability. A similar analysis of distinct bands migrating at approximately 30, 22, and 15 kDa in the Cashewgurt sample did not generate good peptide matches and the identity of those proteins could not be reliably assigned.

### 2.3. Cashew Allergen Peptides in Cashewgurt

To assess the presence of peptides derived from cashew allergens in the yogurt sample, peptides detected by LC–MS/MS analysis of a normalized amount of protein from trypsinized yogurt protein were compared to those observed from trypsinized whole cashew extract. Thirty six percent of Ana o1, 87% of Ana o2 and 60% of Ana o3 sequences were observed in the trypsinized total cashew nut extract. By comparison, a reduction in Ana o 1 (11%) and Ana o 2 (70%) peptide sequences was detected while a slight increase in Ana o 3 (65%) peptides was observed in the Cashewgurt samples (Table 2). This represents 3, 20, and 11 linear peptides from Ana o 1, Ana o 2, and Ana o 3, respectively, observed in the fermented Cashewgurt extracts in comparison with 11, 47, and 11 linear peptides observed in the from the cashew nut extract.

### 2.4. Cashewgurt and Cashew Nut Extract Antibody–Allergen Binding Comparison

**Figure 2:**
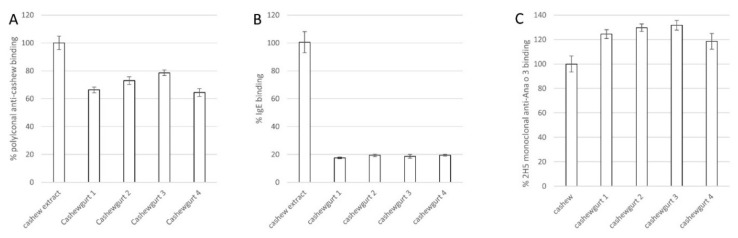
Antibody binding to cashew allergens in soluble Cashewgurt protein**.** Direct ELISA comparison of cashew allergen binding to soluble Cashewgurt protein with rabbit polyclonal anti-cashew (**A**), human cashew-allergic IgE (**B**), and 2H5 monoclonal anti-Ana o 3 (**C**) antibodies; bar graphs represent mean percentage values as compared to antibody binding to cashew nut extract from at least four assays ± percent standard deviation.

ELISA was used to evaluate cashew allergen antibody binding from four independent samples of Cashewgurt. Each of the yogurt samples had reduced rabbit IgG polyclonal anti-cashew antibody binding (Figure 2A). The rabbit IgG polyclonal antibody has been previously shown to recognize the Ana o 1, Ana o 2, and Ana o 3 cashew allergens [16]. Overall, the average of the polyclonal antibody binding to the yogurt samples was reduced to 71% of the binding to cashew nut extract. Similarly, binding to cashew-allergic volunteer IgE was reduced in each Cashewgurt sample (Figure 2B). Overall, IgE binding to soluble Cashewgurt protein was reduced to 19% of the binding observed in whole cashew nut extract. In contrast, relative binding of the murine monoclonal IgG anti-Ana o 3 antibody (2H5) to the Cashewgurt samples was increased (Figure 2C). Overall, mean 2H5 antibody binding was 26% higher in the Cashewgurt sample compared to the cashew nut extract.

## 3. Discussion

When compared to dairy-based yogurt, the Cashewgurt evaluated here had some similar physicochemical properties including pH and viscosity. In contrast, the Cashewgurt had a different color and relatively lower titratable acidity (TA). Cow milk contains approximately 4.6% lactose, but lactose is not naturally present in cashew nuts. The lactobacilli and *S. thermophilus* would not be expected to produce TA from lactose fermentation in the Cashewgurt, and this might explain the lower TA in the Cashewgurt. The lack of lactose did not prevent accumulation of LAB to probiotic levels. Tharmaraj and Shah (2003) reported that probiotic health benefits from consuming LAB required counts of at least 10^6^ CFU/mL [21]. Thus, the lactobacilli and *S. thermophilus* content of the Cashewgurt characterized here may be considered beneficial to health.

Physical, thermal, and enzymatic processing of nut allergens can be effective in reducing IgE binding to allergens [14,15,16,17,22,23]. The findings presented here demonstrate that fermentation by LAB can also reduce IgG and IgE antibody binding to cashew allergens. We cannot rule out that some of the reduced antibody binding to cashew nut allergens could also be due in part to other processing steps not specifically tested here. Further, while there could be some matrix effect in the Cashewgurt samples that is absent in the whole cashew nut extract sample resulting in altered ELISA results, this seems unlikely based upon the different results observed using human IgE, rabbit polyclonal IgG, and mouse monoclonal IgG. In addition, the ELISA results are consistent with the observations on the SDS-PAGE gels. SDS-PAGE analysis indicated that Ana o 1 and Ana o 2 were metabolized well by the LAB into fragments, suggesting that conformational epitopes of Ana o 1 and Ana o 2 are likely destroyed resulting in a significant reduction in IgE binding. Further, fewer unique linear peptides derived from Ana o 1 and Ana o 2 were detected in the Cashewgurt samples, again indicating that Ana o 1 and Ana o 2 were metabolized well by the LAB. However, a large portion of the Ana o 2 protein was still observed in the mass spectrometric data from the soluble Cashewgurt protein, indicating that a number of linear Ana o 2 peptides remain. Although there was a reduced level of intact cashew-allergic proteins, it is likely not sufficient to render Cashewgurt safe for cashew-allergic patients to consume. Importantly, as oral immunotherapy for nut allergy is becoming more accepted, there may be patients who would like to use Cashewgurt for their treatment source of cashew, but this would be premature and potentially dangerous. Allergen recognition patterns and individual IgE-binding sequences can be biased towards one cashew allergen or be equally distributed among each of them. For example, Ana o 2, an immuno-dominant cashew allergen, could account for much of the IgE signal from the sera used in this study, thus the relatively larger reduction in IgE binding compared to the other antibodies. Evaluation with sera from individuals that more robustly recognize Ana o 3 might be expected to indicate that IgE binding was retained after fermentation. In support of this, Ana o 3 was detected by immunoblot with anti-cashew polyclonal antibody and identified by mass spectrometry from an SDS-PAGE band migrating at the expected size of the intact protein. In addition, several peptides matching Ana o 3 sequences were identified from slightly faster migrating bands below the 10 kDa marker. These faster migrating bands suggest that some portion of Ana o 3 has been at least partially metabolized. Consistent with the preservation of Ana o 3, binding of Cashewgurt samples by the 2H5 monoclonal antibody, which recognizes Ana o 3, was not reduced. However, the 2H5 antibody was generated using a linear Ana o 3 peptide sequence, and this antibody has a lower K_D_ for the linear peptide compared to the native protein [24]. These findings reinforce the idea that there may have been at least some proteolysis or unfolding of the native Ana o 3 protein resulting in increased exposure of the linear 2H5 epitope for binding. Alternatively, because the samples were normalized by total protein content, the slight increase in binding of the 2H5 antibody in the Cashewgurt samples could be due to a relative increase in intact Ana o 3 content within the sample.

Protein degradation is an essential process during LAB fermentation. In this study, Ana o3 was more resistant to degradation than either Ana o 1 or Ana o2. This is consistent with several publications, indicating the relative stability of 2S albumins such as Ana o 3 to processing steps [13,14,15,22,23].

*L. lactis* is the primary LAB used for milk fermentation and the production of sour cream, kefir, and cheese. Identification and characterization of the proteolytic enzymes in LAB used during fermentation might enable improved degradation of cashew or other nut allergens during fermentation. Alternatively, evaluation of other LAB (such as *L. helveticus*) or LAB with engineered peptidases during fermentation of cashew nuts or cashew nut flour may provide useful insights.

Current peanut oral immunotherapy (OIT) often uses defatted peanut flour, but other forms of peanuts or peanut-containing foods are also used. The success of peanut OIT regimens varies; in most cohorts, approximately 80% of subjects attain desensitization and some level of long-term protection against accidental exposures, but adverse reactions occur during the desensitization process [25,26,27]. Adverse reactions including abdominal pain, nausea, and vomiting are cited as primary reasons for voluntary discontinuation of OIT.

Presentation of food allergens in the context of certain bacteria or bacterial metabolites, such as SCFAs generated during fermentation, may bias the immune response to downregulate TH2 activity while increasing TH1 regulatory activity towards tolerance [28,29]. For example, in mouse models, the presence of LAB may act as a prophylactic and reduce the severity of allergy symptoms [30,31]. Similarly, combining peanut OIT and LAB probiotics resulted in a long-term reduction in allergic symptoms in human volunteers even after treatment termination [32,33], although the outcome was not superior to OIT without a probiotic. The mechanism by which bacterial metabolites, such as those from LAB, lead to the downregulation of inflammatory pathways likely involves G protein-coupled receptors that sense these metabolites and alter immune cell behavior [34].

The use of attenuated bacteria and viruses as vaccines is a widely accepted strategy to mount a preventive immune response to protect against infectious agents, but vaccines do not always provide complete protection due to strain variations in important immunogenic factors. Analogously, it remains to be determined if OIT, which is thought to re-route an existing immune response, with food options containing attenuated or missing allergens/epitopes, such as the fermented Cashewgurt described here, will provide absolute protection against accidental exposure to foods containing a full complement of unmodified allergen content. In support of this, there is evidence from several patients that OIT using cooked egg or wheat does not provide complete protection because when patients encounter a less thoroughly cooked or raw food during an accidental exposure they react (unpublished results R.L. Wasserman).

Fermentation of cashew nut flour by lactic acid bacteria to produce the commercial Cashewgurt evaluated here reduces IgG and IgE antibody binding to cashew allergens. The data presented here, however, indicate that immuno-reactive fragments of Ana o 1, Ana o 2, and Ana o 3 survive commercial fermentation. While intact Ana o 1 and Ana o 2 were not observed by SDS-PAGE, long stretches of tryptic peptides from Ana o 2 and Ana o 3 were observed. Notably, the SDS-PAGE, LC–MS/MS, and ELISA data presented here with the 2H5 anti-Ana o 3 antibody indicate that the Ana o 3 content of the Cashewgurt is similar to that of untreated cashew extract. Overall, the data lead to the conclusion that Ana o 3, an immuno-dominant cashew allergen strongly correlating with systemic allergic reactions, survives LAB fermentation relatively intact. Continued evaluation of fermentation conditions including bacterial strain selection, media content, and fermentation times could result in cashew nut-based yogurt with a rationally engineered allergen content.

## 4. Materials and Methods

### 4.1. Materials

Containers of Forager Project Plain Cashewgurt were purchased from local grocery stores in the New Orleans area. “Ready-to-eat” cashew nuts were purchased from nuts.com (https://nuts.com/). Phosphate-buffered saline (PBS, pH 7.4), agar, nalidixic acid, neomycin sulfate, lithium chloride, and paromomycin sulfate were obtained from Sigma Aldrich (St. Louis, MO, USA). Novex 10–20% tricine protein gels for SDS-PAGE, SimplyBlue SafeStain, bovine serum albumin (BSA, fraction V), and flat-bottom clear-well MaxiSorp 96-well plates were purchased from ThermoFisher Scientific (Grand Island, NY, USA) and Precision Plus Protein Dual Color molecular weight standards from Bio-Rad (Hercules, CA, USA). The rabbit polyclonal anti-cashew sera have been previously demonstrated to recognize Ana o 1, 2, and 3, as well as several other unidentified cashew nut proteins [16]. The 2H5 monoclonal anti-Ana o 3 peptide antibody has been previously characterized [24]. Blood samples from 12 cashew-allergic donors were obtained from PlasmaLab Int. (Everett, WA, USA). Streptavidin and secondary antibodies to rabbit or mouse IgG labeled with IRDyes (IRDye680RD or IRDye800CW) were from LI-COR Biosciences (Lincoln, NE, USA) and biotinylated mouse anti-human IgE from SouthernBiotech (Birmingham, AL, USA). Sequencing-grade, modified trypsin was purchased from Promega (Madison, WI, USA) and used according to the manufacturer’s instructions.

### 4.2. Microbiological Analyses

For each microbiological analysis described below, 11 g of Cashewgurt was suspended in 99 mL of 0.1% peptone water (Difco Laboratories Inc, Franklin Lakes, NJ, USA), and serial dilutions of 10^−1^ to 10^−9^ were pour plated in duplicate. Lactobacilli were enumerated according to Olson and Aryana (2008) [19]. After, 55 g of Difco Lactobacilli MRS broth (Becton, Dickinson and Co., Franklin Lakes, NJ, USA) was added to 15 g of agar (Sigma-Aldrich Inc., St. Louis, MO, USA) and incorporated into 1 L of distilled water and boiled. The boiled medium was autoclaved at 121 °C for 15 min. Tempered medium was used to pour plates which were incubated anaerobically at 40 °C for 72 h, after which colonies were counted.

Bifidobacteria were enumerated according to the method described by Tharmaraj and Shah (2003) [20]. A liter of MRS NNLP agar contained 52 g of MRS broth, 15 g of agar, 15 mg of nalidixic acid, 100 mg of neomycin sulfate, 3 g of lithium chloride, and 200 mg of paromomycin sulfate. The MRS broth and agar were autoclaved, and the nalidixic acid, neomycin sulfate, lithium chloride and paromomycin sulfate were filter sterilized. The filter-sterilized NNLP was aseptically incorporated into the autoclaved MRS base. Filter-sterilized l-cysteine-HCl (0.05% final concentration) was also added to lower the oxidation-reduction potential of the medium and to enhance the growth of anaerobic bifidobacteria. Pour plates were layered with more MRS NNLP agar soon after solidification. Plates were incubated anaerobically at 37 °C for 72 h.

*Streptococcus thermophilus* (*S. thermophilus*) was enumerated according to Song and Aryana (2014) [21]. Ten (10) grams of tryptone (Becton, Dickinson and Co., Franklin Lakes, NJ, USA), 10 g of sucrose (Amresco, Solon, OH, USA), 5 g of yeast extract (Becton Dickinson and Co., Franklin Lakes, NJ, USA), and 2 g of dipotassium phosphate (Fisher Scientific, Fair Lawn, NJ, USA) were dissolved in 1 L of distilled water. The pH was adjusted to 6.8 + 0.1 with 1 N HCl. Then 6 mL of bromocresol purple (an indictor) and 12 g agar (EMD Chemicals Inc., Gibbstown, NJ, USA) were added. The mixture was boiled and autoclaved at 121 °C for 15 min, tempered and pour plated. Pour plates were incubated aerobically at 37 °C for 24 h and colonies were counted.

Coliform counts were enumerated using coliform petrifilm (3M, St. Paul, MN, USA). Yeast and mold were enumerated using yeast and mold petrifilms (3M, St. Paul, MN, USA). One (1) mL of 10^−1^ dilution was plated on respective petrifilms. Coliform petrifilmswere incubated at 32 °C for 24 h and the yeast and mold petrifilms were incubated at 22 °C for 5 days.

### 4.3. Physicochemical Analyses

Cashewgurt pH was measured using an Oysters Series pH meter (Extech Instruments Corp., Waltham, MA, USA). The pH meter was calibrated using commercial pH 4.00 and 7.00 buffers (Fisher Scientific, Fair Lawn, NJ, USA). The titratable acidity (TA) expressed as percent lactic acid was determined by weighing 9 g of Cashewgurt, adding 5 drops of phenolphthalein indicator and titrating with 0.1 N NaOH to a light pink end point which maintained for 30 s. Viscosity measurements were carried out using a Brookfield DV-II viscometer (Brookfield Engineering Laboratories, Stoughton, MA, USA) with a RV-1 spindle and a helipath stand. The RV-1 spindle was inserted in the yogurt sample at a constant depth of 2 cm from the surface. The helipath was set in downward motion to cut new circular layers at increasing depth of sample at 7 ± 1 °C. The readings were collected with the Wingather software (Brookfield Engineering Laboratories, Stoughton, MA, USA). An average of one hundred data points were taken per sample. The average of 6 samples is reported. Color (L*, a*, b*, C*, h*) was measured using a colorimeter Mini Scan XE Plus (Hunter Lab, Reston, VI, USA). Before measurements, the colorimeter was calibrated with black and white tiles. Readings were taken under D 65 illumination, 10° observer in the reflected mode. An average of five readings were taken per sample. Table 1 data represent the average of six samples with ± standard deviations of the various physicochemical attributes of Cashewgurt.

### 4.4. Cashew Extract Preparation

Cashew nuts were frozen, ground to homogeneity in a coffee grinder, and defatted using a Soxhlet extraction apparatus with petroleum ether overnight. The defatted cashew flour was dried overnight to remove residual petroleum ether, and 50 g was added to 500 mL of phosphate-buffered saline (PBS, pH 7.4). Following 2 h of stirring at room temperature, the solubilized cashew nut extract was centrifuged at 12,000× rpm and filtered through a 0.22 µM PES filter. The filtered extract was aliquoted and frozen at −80 °C for storage.

### 4.5. Sodium Dodecyl Sulfate–Polyacrylamide Gel Electrophoresis (SDS-PAGE) and Immunoblot

The protein content of Cashewgurt was evaluated from at least four different independent samples with tricine-buffered gels by SDS-PAGE. Soluble Cashewgurt protein was collected by gently mixing samples with PBS at a 1:1 ratio (*w*/*v*) for 5 min at room temperature. The Cashewgurt–PBS mixture was centrifuged in a microcentrifuge at 14,000× rpm for 5 min. The protein concentration of the cleared supernatant was measured by absorbance at 280 nm (Nanodrop, ThermoFisher Scientific, Grand Island, NY, USA). Normalized protein content (30 µg) of whole cashew nut extract or soluble Cashewgurt samples was loaded into gel wells and electrophoresed on Novex 10–20% tricine protein gels at 120 V for 90 min in an XCell SureLock Mini gel rig (ThermoFisher Scientific, Grand Island, NY, USA). Protein content was visualized by staining with SimplyBlue SafeStain and scanning on an Odyssey Clx instrument (LI-COR Biosciences, Lincoln, NE, USA). For immunoblot, electrophoresed proteins were transferred to nitrocellulose using the iBlot gel transfer system (Invitrogen/ThermoFisher Scientific, Waltham MA, USA). Blots were blocked in PBS with 0.1% Tween-20 (PBST) and 2% (*w*/*v*) non-fat dry milk for 30 min at room temperature. The rabbit anti-cashew antibody (1:5000 in PBST) was added to the blot and incubated for 1 h at room temperature. Primary antibody was removed by washing three times for 5 min with 5 mL of PBST. Donkey anti-rabbit IRdye-6801 (1:10,000 in PBST) was added and blots were incubated at room temperature for 30 min. The membrane was washed three times as above to remove excess secondary antibody and visualized using an Odyssey CLx.

### 4.6. Liquid Chromatography–Mass Spectrometry (LC–MS/MS)

Equivalent amounts of soluble Cashewgurt protein and cashew nut extract were trypsinized and analyzed by LC–MS/MS to characterize allergen content. To ensure complete characterization, the results of at least 13 independent sample runs for each of the cashew nut and Cashewgurt protein extracts were merged to show the overall percent of the allergens that were identified (percent coverage). Samples treated with trypsin as previously described [13] were run on an Agilent 6520 Q-TOF LC/MS using a Chip Cube interface with a Large Capacity Chip (II) (Agilent, Santa Clara, CA, USA) and analyzed using Mascot software (Matrix Science, Boston, MA, USA) to determine protein identification and percent of protein coverage.

### 4.7. Enzyme-Linked Immunosorbent Assay (ELISA)

The presence of cashew proteins in Cashewgurt was evaluated using rabbit anti-cashew IgG. Cashew allergens were detected using mouse anti-Ana o 3 IgG or anti-cashew IgE from a pool of cashew-allergic volunteer serum samples in direct ELISA. Soluble protein samples were collected as described above with PBS. Soluble Cashewgurt or cashew extract protein (30 µg/well) was diluted to a final volume of 50 µL with sodium carbonate buffer (0.015 M Na_2_CO_3_, 0.035 M NaHCO_3_, pH 9.6) and added to microtiter plate wells overnight (ON) at 4 °C. After at least 12 h, samples were removed from wells, and plates were incubated for one hour at 37 °C with 50 µL of PBS (10 mM phosphate, 137 mM sodium chloride, pH 7.4) containing 0.1% BSA to block wells. After washing plate wells three times with 100 µL of PBS, 50 µL of primary antibody was diluted in PBS and used at the following dilutions: rabbit polyclonal anti-cashew 1:5000, 2H5 anti-Ana o 3 monoclonal 1:5000, and human sera/IgE 1:5. Primary antibody incubations lasted one hour at 37 °C, and plates were then washed three times with 100 µL of PBS. IRDye-labeled donkey anti-rabbit or donkey anti-mouse secondary antibodies were diluted 1:10,000 in PBS (50 µL final volume per well) and incubated one hour at 37 °C. The biotinylated mouse anti-human IgE was diluted 1:5000 in PBS and plates were incubated for one hour at 37 °C.Following incubation with biotinylated mouse anti-human IgE, plate wells were washed three times with 100 µL of PBS and 50 µL of IRDye-labeled streptavidin diluted 1:10,000 was added to plate wells for 30 min at 37 °C. Plate wells underwent three final washes with 100 µL of PBS prior to scanning with an Odyssey CLx to evaluate antibody binding. ELISA data from at least four assays are presented as the mean percentage compared to antibody binding to complete cashew extract ((IRDye Cashewgurt sample mean / IRDye cashew nut extract mean) × 100) ± percent standard deviation.

## Figures and Tables

**Table 1:** Microbiological and physiochemical properties of Cashewgurt.

Parameter	Average of Six Samples	±Standard Deviation
Lactobacilli count	5.4 × 10^7^ (7.73 Log_10_ CFU/mL)	1.1 × 10^7^
Bifidobacteria count	2.5 × 10^4^ (4.40 Log_10_ CFU/mL)	3.0 × 10^4^
*Streptococcus thermophilus* count	2.6 × 10^7^ (7.42 Log_10_ CFU/mL)	9.8 × 10^6^
Coliform count	<10	-
Yeast and mold count	<10	-
pH	4.46	0.11
% TA	0.53	0.02
Viscosity (cP)	13649	2938
L*	84.69	0.47
a*	0.73	0.15
b*	8.89	0.53
C*	8.92	0.52
h*	85.23	1.18

% TA is titratable acidity; L* represents the lightness–darkness axis and ranges from 100 (white) to 0 (black); a* represents the red–green axis, with + a* being red while a* represents green; b* represents the yellow–blue axis, with +b* being yellow while –b* represents blue; C* represents the chroma or color intensity; h* is the hue angle.

**Table 2:** Ana o 1, Ana o 2, and Ana o 3 peptides observed by LC–MS/MS in soluble Cashewgurt protein.

Ana o 1 (Vicilin-Like Protein)	
Cashew nut 1	MGPPTKFSFSLFLVSVLVLCLGFALA***KIDPELK***QCKHQCKVQR***QYDEQQKEQCVK***ECEKY 60
Cashewgurt 1	MGPPTKFSFSLFLVSVLVLCLGFALA***KIDPELK***QCKHQCKVQR***QYDEQQKEQCVK***ECEKY 60
Cashew nut 61	YKEKKGREREHEEEEEEWGTGGVDEPSTHEPAEK***HLSQCMR***QCERQEGGQQKQLCRFRCQ 120
Cashewgurt 61	YKEKKGREREHEEEEEEWGTGGVDEPS***THEPAEKHLSQCMR***QCERQEGGQQKQLCRFRCQ 120
Cashew nut 121	ERYKKERGQHNYKREDDEDEDEDEAEEEDENPYVFEDEDFTTKVKTEQGKVVLLPKFTQK 180
Cashewgurt 121	ERYKKERGQHNYKREDDEDEDEDEAEEEDENPYVFEDEDFTTKVKTEQGKVVLLPKFTQK 180
Cashew nut 181	SKLLHALEKYR***LAVLVANPQAFVVPSHMDADSIFFVSWGR***GTITKILENKRESINVR***QGD*** 240
Cashewgurt 181	SKLLHALEKYRLAVLVANPQAF***VVPSHMDADSIFFVSWGR***GTITKILENKRESINVRQGD 240
Cashew nut 241	***IVSISSGTPFYIANNDENEKLYLVQFLRPVNLPGHFEVFHGPGGENPESFYRAFSWEILE*** 300
Cashewgurt 241	IVSISSGTPFYIANNDENEKLYLVQFLRPVNLPGHFEVFHGPGGENPESFYR***AFSWEILE*** 300
Cashew nut 301	***AALKTSKDTLEK***LFEKQDQGTIMKASKEQIRAMSRRGEGPK***IWPFTEESTGSFK***LFKKDP 360
Cashewgurt 301	***AALK***TSKDTLEKLFEKQDQGTIMKASKEQIRAMSRRGEGPKIWPFTEESTGSFKLFKKDP 360
Cashew nut 361	SQSNK***YGQLFEAERIDYPPLEK***LDMVVSYANITK***GGMSVPFYNSR***ATKIAIVVSGEGCVE 420
Cashewgurt 361	SQSNKYGQLFEAERIDYPPLEKLDMVVSYANITKGGMSVPFYNSRATKIAIVVSGEGCVE 420
Cashew nut 421	IACPHLSSSKSSHPSYKKLRARIRKDTVFIVPAGHPFATVASGNENLEIVCFEVNAEGNI 480
Cashewgurt 421	IACPHLSSSKSSHPSYKKLRARIRKDTVFIVPAGHPFATVASGNENLEIVCFEVNAEGNI 480
Cashew nut 481	RYTLAGKKNIIKVMEKEAKELAFK***MEGEEVDKVFGKQDEEFFFQGPEWR***KEKEGRADE 538
Cashewgurt 481	RYTLAGKKNIIKVMEKEAKELAFKMEGEEVDKVFGKQDEEFFFQGPEWRKEKEGRADE 538
Ana o 2 (legumin-like protein)	
Cashew nut 1	LSVCFLILFHG*CLA**SRQEWQQQDECQIDRLDALEPDNRVEYEAGTVEAWDPNHEQFRCAG** 60*
Cashewgurt 1	LSVCFLILFHGCLASRQEWQQQDECQIDR***LD******ALEPDNRVEYEAGTVEAWDPNHEQFRCAG*** 60
Cashew nut 61	***VALVRHTIQPNGLLLPQYSNAPQLIYVVQGEGMTGISYPGCPETYQAPQQGR***QQGQSGRF 120
Cashewgurt 61	***VALVRHTIQPNGLLLPQYSNAPQLIYVVQGEGMTGISYPGCPETYQAPQQGR***QQGQSGRF 120
Cashew nut 121	QDRHQKIRRFR***RGDIIAIPAGVAHWCYNEGNSPVVTVTLLDVSNSQNQLDRTPRKFHLAG*** 180
Cashewgurt 121	QDRHQKIRRFR***RGDI******IAIPAGVAHWCYNEGNSPVVTVTLLDVSNSQNQLDR***TPR***KFHLAG*** 180
Cashew nut 181	***NPKDVFQQQQQHQSRGRNLFSGFDTELLAEAFQVDER***LIKQLK***SEDNRGGIVKVKDDELR*** 240
Cashewgurt 181	***NPKDVFQQQQQHQSRGRNLFSGFDTELLAEAFQVDER***LIKQLKSEDNRGGIVKVKDDELR 240
Cashew nut 241	VIRPSRSQSER***GSESEEESEDEKRR***WGQR***DNGIEETICTMRLKENINDPARADIYTPEVG*** 300
Cashewgurt 241	VIRPSRSQSER***GSES******EEESEDEK***RRWGQRDN***GIEETICTMRLKENINDPARADIYTPEVG*** 300
Cashew nut 301	***RLTTLNSLNLPILKWLQLSVEKGVLYKNALVLPHWNLNSHSIIYGCKGKGQVQVVDNFGN*** 360
Cashewgurt 301	***RLTTLNSLNLPILKWLQLSVEK***GVLYK***NALVLPHWNLNSHSIIYGCKGKGQVQVVDNFGN*** 360
Cashew nut 361	***RVFDGEVREGQMLVVPQNFAVVKR***AR***EERFEWISFKTNDRAMTSPLAGRTSVLGGMPEEV*** 420
Cashewgurt 361	***RVFDGEVREGQMLVVPQNFAVVK***RAREER***FEWISFK***TNDR***AMTSPLAGRTSVLGGMPEEV*** 420
Cashew nut 421	***LANAFQISREDARKIKFNNQQTTLTSGESSHHMRD***DA 457
Cashewgurt 421	***LANAFQISR***EDARKIKFNNQQTTLTSGESSHHMRDDA 457
Ana o 3 (2S albumin)	
Cashew nut 1	MAKFLLLLSAFAVLLLVANASIYRAIVEVEEDSGR***EQSCQRQFEEQQRF***RNCQR***YVKQEV*** 60
Cashewgurt 1	MAKFLLLLSAFAVLLLVANASIYRAIVEVEEDSGR***EQSCQRQFEEQQR***FRNCQR***YVKQEV*** 60
Cashew nut 61	***QR***GGRYNQR***QESLRECCQELQEVDRRCRCQNLEQMVRQLQQQEQIKGEEVRELYETASEL*** 120
Cashewgurt 61	***QR***GGRYNQRQESLR***ECCQELQEVDRR***CR***CQNLEQMVRQLQQQEQIKGEEVRELYETASEL*** 120
Cashew nut 121	***PRICSISPSQGCQFQSSY*** 138
Cashewgurt 121	***PRICSISPSQGCQFQSSY*** 138

Mass spectrometric protein coverage maps of cashew allergen peptides from trypsin-treated cashew nut and Cashewgurt soluble protein extracts; residues in bold italics were observed by mass spectrometry, while underlined residues indicate published IgE-binding epitopes.
